# Using the Technology Acceptance Model to Characterize Barriers and Opportunities of Telemedicine in Rural Populations: Survey and Interview Study

**DOI:** 10.2196/35130

**Published:** 2022-04-15

**Authors:** Bree Holtz, Katharine Mitchell, Kelly Hirko, Sabrina Ford

**Affiliations:** 1 Department of Advertising and Public Relations College of Communication Arts & Sciences Michigan State University East Lansing, MI United States; 2 Department of Epidemiology and Biostatistics College of Human Medicine, Traverse City Campus Michigan State University Traverse City, MI United States; 3 Department of Obstetrics, Gynecology & Reproductive Biology College of Human Medicine Michigan State University East Lansing, MI United States

**Keywords:** telehealth, technology acceptance model, pilot study, rural, Michigan, health care access, telemedicine, phone interviews, paper surveys

## Abstract

**Background:**

Health care access issues have long plagued rural Americans. One approach to alleviating the challenges and poor health outcomes for rural individuals is through the use of telemedicine, sometimes called telehealth. It is important to understand factors that may be related to telemedicine adoption or nonadoption, particularly in underserved rural settings.

**Objective:**

This pilot study examines telemedicine perceptions among rural, underserved populations using the Technology Acceptance Model, which serves as a framework to explore the adoption of telemedicine services by those who have used it. This study also explores the differences between user and nonuser perceptions of telemedicine.

**Methods:**

Paper surveys and phone interviews were conducted in rural Northern Lower Michigan.

**Results:**

Perceived usefulness and perceived ease of use explained 91% of the variability in attitude toward telemedicine (*R*^2^=0.91; *F*_1,15_=73.406; *P*<.001). Ease of use was a significant predictor (mean 2.36, SD 1.20; *P*<.001), but usefulness (mean 3.16, SD 0.81; *P*=.20) was not. Furthermore, there were significant differences in individual perception of telemedicine between users and nonusers. For example, nonusers believed they would receive better care in person (users: mean 3.30, SD 1.22; nonusers: mean 1.91, SD 1.14; *F*_1,32_=10.126; *P*=.003). The quantitative findings were reinforced by the qualitative results from the phone interviews.

**Conclusions:**

Overall, the Technology Acceptance Model is an appropriate model to understand the attitudes toward telemedicine that may lead to its adoption by rural Americans.

## Introduction

Americans living in rural areas are more likely to have lower levels of education and live below the average income of the country when compared to their urban counterparts. They are more likely to die from all the leading causes of death in the United States and are more likely to have a disability that leaves them unable to work [1]. Additionally, health care access issues have long plagued the 14.5 million rural Americans [2] who face a severe shortage of health care workers and fewer hospital beds per capita than their urban counterparts [3]. These health care access challenges have been exacerbated during the COVID-19 pandemic. One approach to alleviating these challenges and the related poor health outcomes in rural areas is telemedicine, also often referred to as telehealth. Telemedicine can deliver health care through technologies such as mobile phones or computers. Telemedicine programs can address transportation barriers in geographically dispersed rural regions by allowing patients to remotely connect with their providers and enabling access to specialty providers for consultation from afar [4].

Although telemedicine has been around for decades, the COVID-19 pandemic and the resulting reimbursement policy changes accelerated the service’s growth and awareness among the public [5-7]. In fact, telemedicine has become a viable way—and for some the only way—to see a health care provider. The adoption of telemedicine services during the pandemic has grown exponentially. The Centers for Disease Control and Prevention reported that 43% of health centers could provide telemedicine services in 2019, and 95% of health care centers reported using telemedicine services by April 2020 [8]. Despite expanded reimbursement, the rapid implementation of telemedicine for health care delivery during the pandemic has excluded approximately one-third of rural Americans, who may lack access to the necessary broadband internet needed for telemedicine [9,10]. Other telemedicine barriers affecting rural populations include limited access to technology at home, low digital literacy, and apprehension regarding telemedicine as a viable health service [11].

Therefore, it is important to understand factors related to telemedicine adoption and nonadoption, particularly by individuals in underserved rural settings. For this study, we examined the adoption of telemedicine through the lens of the Technology Acceptance Model (TAM) [12]. This model posits that perceived usefulness and perceived ease of use predict an individual’s attitude toward using the technology. Perceived usefulness has been defined as “the degree to which an individual believes that using a particular system would enhance his or her job performance.” Perceived ease of use is generally defined as how easy a system is to use. Greater perceived usefulness and perceived ease of use for a particular system should be predictive of more favorable attitudes toward the technology or the system itself. Together, these factors determine an individual’s behavioral intentions to use the technology. Several studies have demonstrated that this model has predictive value in the context of health and technology [13-15].

The purpose of this study is to examine telemedicine perceptions among rural, underserved populations (specifically patients) in Northern Lower Michigan using the TAM, which serves as a framework to explore the adoption of telemedicine services by those who have used it. Additionally, the study explores how users and nonusers differ in their perceptions and barriers to using telemedicine, which would provide essential information for the implementation of these services.

## Methods

### Location and Recruitment

**Figure 1:**
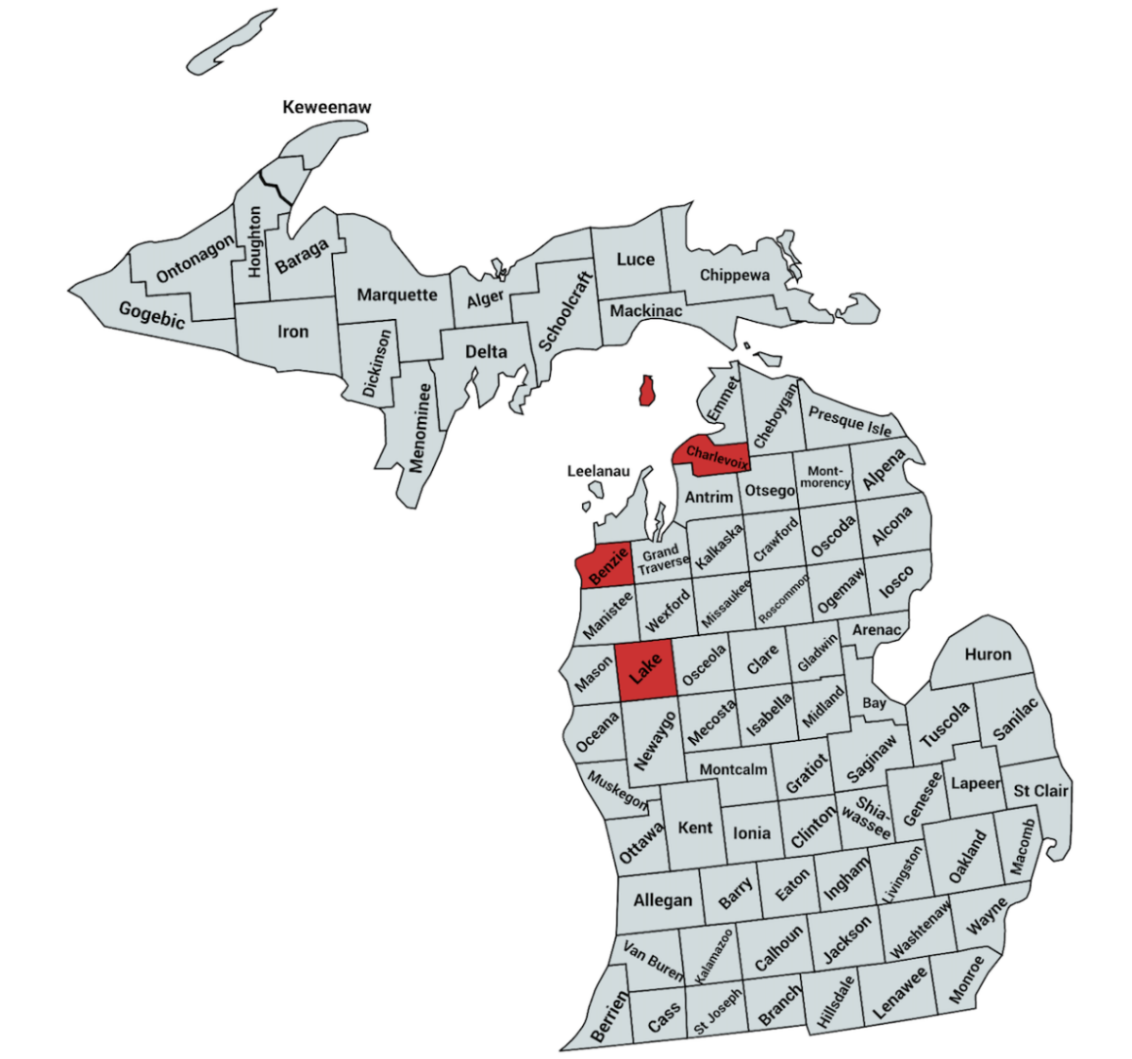
Map of the surveyed areas in red. In Charlevoix County, only Beaver Island was surveyed (map created with MapChart).

In this cross-sectional study, study locations in rural Northern Lower Michigan were selected in collaboration with our community partners and represented rural regions with health care access barriers. Benzie County was selected because it is the smallest county in the state of Michigan and represents a remote rural county with a Rural-Urban Continuum Code (RUCC) of 9 (codes 1-3 denote metropolitan areas, 4-6 suburban, and 7-9 rural) [16]. Its ratio of population to primary care physicians is higher than the state average (1610:1 vs 1030:1) [17]. Lake County (RUCC of 9) was selected because it is the poorest county in Michigan and has the highest ratio of population to primary care physicians in the state of Michigan (11,880:1). Beaver Island, part of Charlevoix County, was selected as it is rural and only accessible by plane or ferry (in warmer weather) and has a RUCC of 7. See Figure 1 for a map of the surveyed areas.

From March to September 2020, researchers provided survey packets to 2 food pantries and an island-based health center in these 3 counties. The food pantry and health center staff handed out the packets to their clients, while keeping a record to ensure only one packet was provided per household. Participants had to be at least 18 years of age and consented to the survey by mailing it back to the researchers. The survey packet included a welcome letter, the paper survey, a self-addressed stamped return envelope, an invitation to participate in a voluntary follow-up phone interview, and a US $2 bill. Paper surveys were used to reach this remote population, comply with COVID-19 research restrictions, and account for uneven technology access among the population.

Respondents were asked if they would be willing to participate in a phone interview to better to understand some of their perceptions regarding telemedicine services. To indicate interest, they completed a contact form to send back with the survey. Interview participants were given a US $20 gift card to a store of their choosing. The phone interviews were conducted by 3 researchers using a semistructured interview guide based on survey responses. All interviews were audio-recorded and transcribed verbatim. The 3 researchers reviewed the recordings and highlighted any commonalities between the respondents.

### Ethics Approval

Michigan State University and Munson Healthcare Institutional Review Boards approved this study (STUDY00004682) and all data collection materials.

### Data Collection

The paper survey in this study was modified based on a survey used in previous studies regarding use, perceptions, and technology access [18]. The survey asked respondents their previous experience using telemedicine services, perceptions of telemedicine (using a Likert-type response scale, from 1=strongly agree to 5=strongly disagree), insurance and employment status, overall health status, technology access, and primary care provider status. Perceived usefulness was measured using 5 statements (eg, “I generally use telemedicine when my provider isn’t open,” “I have used telemedicine because I didn’t want to get infected in the waiting room by other people”). Perceived ease of use was measured using 4 statements (eg, “It was convenient to receive care through telemedicine,” “It was easy to arrange an appointment”). Attitude toward telemedicine was measured using 5 statements (eg, “The quality of care through telemedicine is excellent,” “If I had the opportunity, I would use telemedicine again”). The complete survey can be found in Multimedia Appendix 1.

The semistructured interview guide was developed through a review of the responses to the survey, the TAM constructs, and general use and perceptions of telemedicine.

### Statistical Analysis

Descriptive statistics were used to characterize the study population. The reliabilities of survey questions were reported using Cronbach alpha. In addition, frequencies and percentages were evaluated for variables describing barriers to telemedicine use from nonusers and intentions to use telemedicine from previous users. We conducted a factor analysis using principal component analysis with a varimax rotation. The analysis was able to cluster the TAM variables into the constructs of interest. A multivariate linear regression model was conducted to evaluate the relationship between the continuous TAM variables (ease of use and usefulness) and the outcome of attitude toward telemedicine; assumptions of the linear model were specifically tested. Finally, ANOVA was conducted to compare group differences between users and nonusers on multiple outcomes of interest, including perceptions of in-person care and telemedicine care, the ease of seeing their primary care provider, concerns about insurance, continuity of care, and communication. Statistical significance for all analyses was set as *P*<.05. Data screening was performed for missing data and outliers and the assumptions of multiple regression analysis methods were considered.

## Results

### Respondent Demographics

Characteristics of the 59 survey respondents are shown in Table 1. The majority of respondents lived in Benzie County, Michigan (n=30, 51%), followed by Beaver Island, Michigan (n=16, 27%) and Lake County, Michigan (n=13, 22%). Most respondents identified as female (n=41, 69%) and White (n=41, 69%). A large proportion of respondents indicated having an annual income less than US $20,000 (n=29, 49%) and that they were retired (n=20, 34%) or unable to work (n=10, 17%). In addition, nearly half did not attend college (n=25, 42%). There were 11 (19%) respondents who indicated that they do not have access to the internet in their homes. Overall, 25 (42%) respondents reported having used telemedicine in the past.

**Table 1:** Paper survey respondent demographics.

Variable		All respondents (N=59), n (%)	Users (n=25), n (%)	Nonusers (n=16), n (%)
**Location**				
	Beaver Island, Michigan	16 (27)	7 (28)	5 (31)
	Benzie County, Michigan	30 (51)	15 (60)	8 (50)
	Lake County, Michigan	13 (22)	3 (12)	3 (19)
**Gender identity**				
	Female	41 (69)	18 (72)	11 (69)
	Male	14 (24)	5 (20)	5 (31)
	Prefer not to answer or no response	4 (7)	2 (8)	0 (0)
**Birth year cohort (years)**				
	1934-1940	4 (7)	0 (0)	2 (12)
	1941-1950	9 (15)	3 (12)	3 (19)
	1951-1960	19 (32)	8 (32)	6 (38)
	1961-1970	8 (14)	3 (12)	2 (12)
	1971-1980	8 (14)	6 (24)	1 (6)
	1981-1990	5 (8)	3 (12)	0 (0)
	Prefer not to answer or no response	6 (10)	2 (8)	2 (12)
**Race and ethnicity**				
	American Indian, Alaska Native, or Native Hawaiian	3 (5)	2 (8)	0 (0)
	Black or African American	3 (5)	0 (0)	1 (6)
	White	41 (69)	19 (76)	10 (62)
	Prefer not to answer or no response	12 (20)	4 (16)	5 (31)
**Household income (US $)**				
	<20,000	29 (49)	12 (48)	6 (38)
	20,000-34,999	12 (20)	6 (24)	5 (31)
	35,000-49,999	4 (7)	2 (8)	0 (0)
	50,000-74,999	2 (3)	0 (0)	1 (6)
	75,000-99,999	1 (2)	0 (0)	1 (6)
	Prefer not to answer or no response	11 (19)	5 (20)	3 (19)
**Education**				
	No schooling completed	1 (2)	0 (0)	0 (0)
	Grades 1 through 11	2 (3)	0 (0)	0 (0)
	Regular high school diploma	16 (27)	6 (24)	4 (25)
	GED^a^ or alternative credential	6 (10)	3 (12)	1 (6)
	Some college credit, but less than 1 year of college	5 (8)	2 (8)	3 (19)
	1 or more years of college credit, no degree	10 (17)	3 (12)	2 (12)
	Associates degree (eg, AA, AS)	3 (5)	2 (8)	1 (6)
	Bachelor’s degree (eg, BA, BS)	4 (7)	2 (8)	2 (12)
	Master’s degree (eg, MA, MS, MEng, MEd, MSW, MBA)	6 (10)	3 (12)	3 (19)
	Doctorate degree (eg, PhD, EdD)	1 (2)	1 (4)	0 (0)
	Prefer not to answer or no response	5 (8)	3 (12)	0 (0)
**Current employment status**				
	Employed for wages	7 (12)	5 (20)	1 (6)
	Self-employed	3 (5)	0 (0)	2 (12)
	Out of work and looking for work	3 (5)	1 (4)	1 (6)
	Out of work, but not currently looking for work	2 (3)	2 (8)	0 (0)
	A homemaker	2 (3)	0 (0)	1 (6)
	Retired	20 (34)	5 (20)	9 (56)
	Unable to work	10 (17)	3 (12)	1 (6)
	Other	7 (12)	6 (24)	0 (0)
	Prefer not to answer or no response	5 (8)	3 (12)	1 (6)
**Internet access**				
	Via cellular data plan for a smartphone/other mobile device	31 (53)	18 (72)	8 (50)
	Via broadband internet	23 (39)	10 (40)	7 (44)
	Via satellite internet	9 (15)	3 (12)	4 (25)
	Via dial-up internet	2 (3)	1 (4)	1 (6)
	Don’t know	2 (3)	0 (0)	0 (0)
	I do not have access to the internet	11 (19)	2 (8)	2 (12)

^a^GED: general educational development.

### Quantitative Results

#### Reliability

The reliabilities of survey statements on 3 variables were reported: perceived usefulness (Cronbach α=.73), perceived ease of use (Cronbach α=.87), and attitude toward telemedicine (Cronbach α=.93).

#### TAM Results

Perceived usefulness and perceived ease of use explained 91% of the variability in attitude toward telemedicine (*R*^2^=0.91; *F*_1,15_=73.406; *P*<.001). Ease of use was a significant predictor of attitude toward telemedicine (mean 2.36, SD 1.20; *P*<.001), but usefulness (mean 3.16, SD 0.81; *P*=.20) was not. See Table 2 for the full regression analysis.

**Table 2:** Regression analysis: usefulness and ease of use as a predictor of attitude toward telemedicine.

Effect	Unstandardized B	SE	Standardized β	*t* value (*df*)	*P* value
Usefulness	–0.174	0.128	–.119	–1.357 (17)	.20
Ease of use	0.997	0.087	.999	11.404 (17)	<.001

#### User and Nonuser Perceptions

Significant differences were observed between users and nonusers on their perceptions of in-person care compared to telemedicine. When comparing previous users of telemedicine to nonusers, the nonusers believed they would receive better care in person compared to telemedicine (users: mean 3.30, SD 1.22; nonusers: mean 1.91, SD 1.14; *F*_1,32_=10.126; *P*=.003). Nonusers also believed that health care providers would not be as caring via telemedicine (users: mean 4.09, SD 1.15; nonusers: mean 2.91, SD 1.04; *F*_1,31_=8.199; *P*=.007). Finally, the results demonstrated significant differences between users and nonusers on worries about continuity of care (users: mean 4.05, SD 0.95; nonusers: mean 3.00, SD 0.78; *F*_1,31_=9.957; *P*=.004), with nonusers having more worries regarding the continuity of care than past users of telemedicine. There were no significant differences between users and nonusers on the ease of seeing their primary care provider (*P*=.26), concerns about insurance (*P*=.31), concerns about experiencing worse communication (*P*=.74), or worries about their primary care provider receiving their information (*P*=.18). See Table 3 for the full results and statistics.

**Table 3:** Means, standard deviations, and 1-way ANOVA in outcomes of users compared to nonusers of telemedicine.

Outcome	Users, mean (SD)	Nonusers, mean (SD)	*F* test (*df*)	*P* value
Easy to see provider	2.78 (1.30)	2.27 (1.01)	1.343 (1,32)	.26
Better care in person	3.30 (1.22)	1.91 (1.14)	10.126 (1,32)	.003
Insurance concerns	3.45 (1.41)	2.91 (1.51)	1.051 (1,31)	.31
Worse communication	3.32 (1.46)	3.50 (1.35)	0.111 (1,30)	.74
Provider not caring	4.09 (1.15)	2.91 (1.04)	8.199 (1,31)	.007
Continuity of care concerns	4.05 (0.95)	3.00 (0.78)	9.957 (1,31)	.004
Concerns about provider not receiving information from visit	3.95 (1.09)	3.45 (0.69)	1.915 (1,31)	.18

### Qualitative Results

Of the survey respondents, 8 individuals indicated interest in participating in the phone interviews and all completed the interviews. The participants were made up of 1 (12%) man and 7 (88%) women, with 5 (62%) participants from Beaver Island, 3 (38%) from Benzie County, and none from Lake County. There were 5 (62%) participants who had used telemedicine in the past. Examples of interview questions are shown in Textbox 1.

Ease of use was reinforced as a key driver of using telemedicine, with the participants indicating that it was often easier to see a provider through telemedicine when compared to the travel time and costs associated with in-person care. Among telemedicine users, positive perceptions of their experiences were expressed in interviews; they would continue to use it and recommend it to others who might be hesitant. Mirroring our quantitative results, trust in their primary care provider was a key theme. It was important for those who have used telemedicine and a reason why some have not. Another barrier mentioned for using telemedicine was the individuals’ access to internet. Many stated that rural internet access was not always stable. There was sometimes only 1 internet provider that was slow, unreliable, or not responsive to their needs. Examples of participant responses are shown in Table 4.

**Sample qualitative interview questions.**

- How does your technology access or connectivity impact your willingness to use telemedicine services?
- Why did you use telemedicine? / Why haven’t you used telemedicine?
- Were you able to have your visit from your home or did you have to go someplace else?
- Describe the process of setting up a telemedicine appointment, from scheduling to the visit.
- How would you describe the communication between you and your provider during the telemedicine visit?
- What are things you liked about your telemedicine visit(s)?
- What are things that you didn’t like about your visit?
- How (if at all) have your past experiences with telemedicine impacted your future use of telemedicine?
- Would you recommend telemedicine to a relative or friend? Why or why not?

**Table 4:** Sample participant responses.

Theme/category	Illustrative quote
Ease of use	I didn't have to go through all the trouble of traveling, taking a whole day to go for a doctor's office visit and then sitting in a waiting room.
Positive perception of telemedicine experience	And I think it's very effective in what it's trying to accomplish. For example, I'm on Beaver Island and I have a teleconference with a doctor off island, the doctor I have on island could be there with me. In other words, we're sharing information. I think it's effective and incredibly beneficial for anybody that has issues that need to be dealt with that way. I like this wonderful service, especially because I live in a remote area of Michigan, and it is a little bit complicated to get to a doctor. For me, telemedicine is really a wonderful service.
Access to internet as a barrier to telemedicine	They're [internet providers] utterly unresponsive. They couldn't care less about the people on the island because they don't have to.
Trust	Probably the connections that they are *[sic]* have with the doctor. If they feel comfortable with their doctor, whether they talking to them over the phone or seeing them face-to-face.

## Discussion

### Principal Findings

This study sought to understand the adoption of telemedicine among people in very rural communities who were accessing food pantries and a community health center amid the COVID-19 pandemic through the lens of the TAM. The results suggested that the TAM is an appropriate model to view the attitudes toward telemedicine that may lead to its adoption by rural Americans. For this population, perceived ease of use was a stronger predictor of telemedicine use than perceived usefulness. Furthermore, there were significant differences in individual perceptions of telemedicine between users and nonusers.

Although perceived usefulness was not significant on its own, it still represents a concept that should be further explored with this population. There are several possible explanations for this finding. For example, a recent study found that some individuals had concerns regarding the privacy and security of telemedicine visits [19]. Perhaps these concerns impacted perceptions of usefulness in this study. Additionally, perceptions of usefulness may be impacted by limited availability and quality of internet-enabled devices, lack of access to high-speed broadband, or health and technology literacy limitations, as participants mentioned these factors in their statements. Another issue noted in other studies is that people like to see their primary care physician in person [18]; more work should be done to explore if this applies for all types of visits.

### Practical Implications

A positive finding from this data was that, once patients have used telemedicine, they generally become more receptive toward it. This mirrors past data regarding users and nonusers of telemedicine during the COVID-19 pandemic [18]. Although this does not suggest that all medical or health visits should be conducted via technology, patients could try telemedicine for simple, quick visits (eg, medication follow-ups, refills) to become more comfortable with technology for future visits. Additionally, these results should have implications on policy changes [20]. For example, in the United States, the Biden infrastructure plan intends to increase access to quality and affordable high-speed internet [21]. Medical schools should offer medical students and residents more opportunities to learn about telemedicine, not just what it is and how it is used, but also how to integrate it into clinical practice, train staff, and prepare patients for their visit. This is especially true for clinics serving primarily older and rural patient populations.

There are limitations of this study that should be mentioned. First, this study examined a specific demographic cohort of mostly low-income, White, older, retired, or disabled participants in Northern Lower Michigan who are traditionally difficult to reach for research, especially during the COVID-19 pandemic. However, it should be noted that gaining insight from this population is important for researchers and policy makers. Although this demographic may not be representative of all rural populations, access issues transcend locations. Further research should be conducted to learn how telemedicine is perceived in various rural populations and locales. Future studies are needed to understand rural individuals’ acceptance of and attitudes toward telemedicine with continued testing and exploration of the TAM within these difficult-to-reach populations. These preliminary data can help other researchers determine if the same constructs remain significant for targeting tailored interventions for their community. This study found that by working with the community and distributing paper surveys through food banks, we were able to access difficult-to-reach populations who provided valuable insights about telemedicine. These findings are useful for future research on this population. Based on these data, we have begun implementing interventions to help increase the use of telemedicine and improve access to health care in these rural areas and throughout other rural regions of Michigan.

### Conclusion

During the COVID-19 pandemic, telemedicine has allowed for continued health care while adhering to strict social distancing policies. This unforeseen experiment has proven that telemedicine has “come of age” after decades of underutilization. This study offers a deeper understanding of attitudes toward and acceptance of telemedicine by vulnerable rural populations in the United States. If telemedicine is used proficiently and consistently, rural populations can gain improved health benefits, which would promote health equity and improve health outcomes.

## Appendix

Multimedia Appendix 1 Paper survey.

## Abbreviations

RUCC: Rural-Urban Continuum Code
TAM: Technology Acceptance Model
