# Supplementary material for: Using the Technology Acceptance Model to Characterize Barriers and Opportunities of Telemedicine in Rural Populations: Survey and Interview Study
Source: JMIR Form Res. 2022 Apr 15;6(4):e35130. doi: 10.2196/35130 (PMC9055487; doi:10.2196/35130)
Supplement: Multimedia Appendix 1 [file formative_v6i4e35130_app1.docx]

**Telemedicine or telehealth** is a way to connect with a doctor or healthcare services through the internet using a computer, tablet or mobile phone.

This survey is going to use the term “telemedicine”.

1. Before this definition was provided, did you know about telemedicine services?

① Yes ***(continue to BLOCK A - question 2)***

② No (***Go to BLOCK D, page 7, question 12)***

**BLOCK A**

1. **Where have you heard about these services? *(check all that apply)***

- Doctor/hospital system
- News
- Insurance company
- Employer
- Internet
- Direct mailer
- Other, please specify________________________
- Don’t remember

1. **When do you think you first became aware of telemedicine services?**

① Over a year ago (before COVID-19)

② 6 months to a year ago (before COVID-19)

③ 3-5 months ago (after COVID-19)

④ 1-2 months ago (after COVID-19)

⑤ Less than a month ago (after COVID-19)

1. **Have you ever used telemedicine?**

① Yes ***(Go to BLOCK B, question 5, page 2)***

② No ***(Go to BLOCK C, question 11, page 6)***

**BLOCK B**

1. **Have you (or your immediate family) used telemedicine before January 2020?**

① Yes, I used telemedicine on or before December 2019

② No, I used telemedicine for the first time in 2020

1. **Why did you (or your immediate family) use these services?**

① I used the services because my primary care provider wasn’t open

(holiday/late night)

② I was told to by my primary care provider

③ I was feeling too sick to leave my house

④ I had to because of COVID-19

⑤ Other, please specify: ____________________

1. **How many times have you (or your immediate family) used telemedicine?**

① 1-5 times

② 6-10 times

③ 11-15 times

④ 16-20 times

⑤ 21+ times

1. **When you have used the telemedicine visits, was the health care provider** **(generally).**

① Physician

② Psychologists

③ Physician Assistant

④ Nurse Practitioner

⑤ Nurse

⑥ Other, please specify:_______________

⑦ Unsure

**9. What service(s) have you used through telemedicine? Check all that apply.**

- “Concierge” services, fee-based
- Medication management/prescription renewal
- Minor urgent care (i.e., pink eye, fevers, etc.)
- Birth control counseling
- Home health care
- Chronic condition management
- Pediatric after-hours needs
- Behavioral/mental health
- Post-hospital discharge
- Post-surgical follow-up
- Other, please specify:________________

**10. Please indicate your level of agreement of each of these statements in regard to your overall experiences of telemedicine.**

|  | ***Strongly Agree*** | ***Agree*** | ***Neutral*** | ***Disagree*** | ***Strongly Disagree*** |
| --- | --- | --- | --- | --- | --- |
| 1. I was able to communicate adequately with the health care provider. | ① | ② | ③ | ④ | ⑤ |
| 1. I was comfortable that the health care provider was able to understand what was bothering me. | ① | ② | ③ | ④ | ⑤ |
| 1. I had difficulty hearing the health care provider over the computer/mobile system. | ① | ② | ③ | ④ | ⑤ |
| 1. I had difficulty seeing the health care provider over the computer/mobile system. | ① | ② | ③ | ④ | ⑤ |
| 1. Telemedicine made it easier to get medical care when I needed it. | ① | ② | ③ | ④ | ⑤ |
| 1. I would have gotten better care if I had seen the health care provider in person. | ① | ② | ③ | ④ | ⑤ |
| 1. Overall, I was very satisfied with telemedicine visits. | ① | ② | ③ | ④ | ⑤ |
| **Please indicate your level of agreement of each of these statements in regard to your overall experiences of telemedicine.** | | | | | |
|  | ***Strongly Agree*** | ***Agree*** | ***Neutral*** | ***Disagree*** | ***Strongly Disagree*** |
| 1. The next time I would prefer to see a health care provider in person despite the possible inconvenience. | ① | ② | ③ | ④ | ⑤ |
| 1. It was easy to arrange an appointment. | ① | ② | ③ | ④ | ⑤ |
| 1. It was convenient to receive care through telemedicine. | ① | ② | ③ | ④ | ⑤ |
| 1. The health care provider dominated the conversation. | ① | ② | ③ | ④ | ⑤ |
| 1. The health care provider spent little time taking my medical history. | ① | ② | ③ | ④ | ⑤ |
| 1. There was less communication with the provider (than I normally receive in person) using telemedicine. | ① | ② | ③ | ④ | ⑤ |
| 1. The health care provider was sensitive to my needs and concerns. | ① | ② | ③ | ④ | ⑤ |
| 1. I am satisfied with the care I received via telemedicine. | ① | ② | ③ | ④ | ⑤ |
| 1. The health care provider who provided me care genuinely seemed to care about me. | ① | ② | ③ | ④ | ⑤ |
| 1. If I had the opportunity, I would use telemedicine again. | ① | ② | ③ | ④ | ⑤ |
| 1. I felt like my privacy was invaded during the telemedicine visit. | ① | ② | ③ | ④ | ⑤ |
| 1. I am worried about the confidentiality of my private information being exchanged through the telemedicine visit. | ① | ② | ③ | ④ | ⑤ |
| 1. I am worried about the continuity of care (i.e., I don’t see my same provider every time). | ① | ② | ③ | ④ | ⑤ |
| 1. I am concerned that my primary care provider will not get my visit information. | ① | ② | ③ | ④ | ⑤ |
| 1. I am concerned that my insurance will not cover my telemedicine visit. | ① | ② | ③ | ④ | ⑤ |
| **Please indicate your level of agreement of each of these statements in regard to your overall experiences of telemedicine.** | | | | | |
|  | ***Strongly Agree*** | ***Agree*** | ***Neutral*** | ***Disagree*** | ***Strongly Disagree*** |
| 1. I generally use telemedicine when my provider isn’t open (e.g., after hours, holidays, etc.). | ① | ② | ③ | ④ | ⑤ |
| 1. I generally use telemedicine when I feel too sick to leave the house. | ① | ② | ③ | ④ | ⑤ |
| 1. I have used telemedicine because I didn’t feel like my condition was too urgent. | ① | ② | ③ | ④ | ⑤ |
| 1. I have used telemedicine because I didn’t want to infect (cold, flu, etc.) other people in a waiting room. | ① | ② | ③ | ④ | ⑤ |
| 1. I have used telemedicine because I didn’t want to get infected in the waiting room by other people (cold, flu, etc.). | ① | ② | ③ | ④ | ⑤ |
| 1. It is easy to get in to see my primary care provider. | ① | ② | ③ | ④ | ⑤ |
| 1. I would recommend telemedicine services to others. | ① | ② | ③ | ④ | ⑤ |
| 1. The quality of care through telemedicine is excellent. | ① | ② | ③ | ④ | ⑤ |
| 1. I am worried about the accuracy of the information from the telemedicine health care provider. | ① | ② | ③ | ④ | 🖑 |

**Block C**


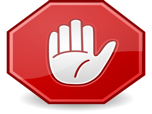


[This Photo](https://commons.wikimedia.org/wiki/File:Dialog-stop-hand.svg) by Unknown Author is licensed under [CC BY-SA](https://creativecommons.org/licenses/by-sa/3.0/)

**[IF YOU ANSWERED *BLOCK B* – SKIP TO BLOCK D, QUESTION 12, PAGE 7.)**

**11. Indicate how much you agree with each statement.**

| ***I have not used telemedicine because…*** | ***Strongly Agree*** | ***Agree*** | ***Neutral*** | ***Disagree*** | ***Strongly Disagree*** |
| --- | --- | --- | --- | --- | --- |
| 1. It is easy to get into my primary care provider. | ① | ② | ③ | ④ | ⑤ |
| 1. I prefer going to walk-in clinics. | ① | ② | ③ | ④ | ⑤ |
| 1. I am unsure if my insurance covers these visits. | ① | ② | ③ | ④ | ⑤ |
| 1. I am worried about the ability to communicate adequately with the health care provider. | ① | ② | ③ | ④ | ⑤ |
| 1. I think I would get better care in person. | ① | ② | ③ | ④ | ⑤ |
| 1. I don’t have very good internet. | ① | ② | ③ | ④ | ⑤ |
| 1. I am not technologically savvy enough to use telemedicine services. | ① | ② | ③ | ④ | ⑤ |
| 1. I don’t know how to get telemedicine care. | ① | ② | ③ | ④ | ⑤ |
| 1. I would get better care if I see my provider in person. | ① | ② | ③ | ④ | ⑤ |
| 1. I worry about the quality of communication with a provider using telemedicine. | ① | ② | ③ | ④ | ⑤ |
| 1. I worry that the health care provider will not be sensitive to my needs. | ① | ② | ③ | ④ | ⑤ |
| 1. I worry about confidentiality of my private information being exchanged through telemedicine. | ① | ② | ③ | ④ | ⑤ |
| 1. I worry about the continuity of care (i.e., I don’t see the same provider every time). | ① | ② | ③ | ④ | ⑤ |
| 1. I am concerned that my primary care provider would not get my visit information. | ① | ② | ③ | ④ | ⑤ |
| 1. I don’t know how to find telemedicine services. | ① | ② | ③ | ④ | ⑤ |
| 1. I don’t have the technology needed for telemedicine visits. | ① | ② | ③ | ④ | ⑤ |
| 1. I think it would take longer to have a visit over telemedicine than in person. | ① | ② | ③ | ④ | ⑤ |

**BLOCK D**

***[All survey respondents please answer the following questions.]***

1. **What services do you think would be good use for telemedicine?**

Some examples could include prescription renewal, minor urgent care (i.e., pink eye, fevers, etc.), birth control counseling, home health care, chronic condition management, pediatric after-hours needs, behavioral/mental health, post-hospital discharge, post-surgical follow-up, etc.

1. **What services do you think would NOT be good use for telemedicine?**
2. **What services would YOU be most likely to use?**
3. **In general, would you say that your health is**

① Excellent

② Very good

③ Good

④ Fair

⑤ Poor

1. **Do you have a primary care provider?**

① Yes

② No

1. **I have access to the Internet using *(check all that apply):***

- Cellular data plan for a smart phone or other mobile device
- Broadband (high speed) internet service such as cable, fiber optic, or DSL service
- Satellite internet service
- Dial-up Internet service
- Don’t know
- I do NOT have access to the internet at home

1. **What technology do you currently own? *(check all that apply)***

- Desktop computer
- Laptop
- Tablet
- E-reader
- Smart watch (Apple watch, Fitbit, etc.)
- Cell phone

1. **To which gender identify do you most identify?**

① Female

② Male

③ Not listed, ______

④ Prefer not to answer

1. **What year were you born?**

|  |  |  |  |
| --- | --- | --- | --- |

1. **Are you a caregiver to (*check all that apply)*:**

- Adult parents
- Adult siblings
- Child(ren)

1. **What is your household income?**

① Less than $20,000

② $20,000 to 34,999

③ $35,000 to 49,999

④ $50,000 to 74,999

⑤ $75,000 to $99,999

⑥ Over $100,000

⑦ Prefer not to answer

1. **What is the highest degree or level of school you have completed?**

① No schooling completed

② Grades 1 through 11

③ 12th grade—no diploma

④ Regular high school diploma

⑤ GED or alternative credential

⑥ Some college credit, but less than 1 year of college

⑦ 1 or more years of college credit, no degree

⑧ Associates degree (for example: AA, AS)

⑨ Bachelor’s degree (for example: BA. BS)

⑩ Master’s degree (for example: MA, MS, MEng, MEd, MSW, MBA)

^11^ Professional degree beyond bachelor’s degree (for example: MD, DDS, DVM, LLB, JD)

^12^ Doctorate degree (for example, PhD, EdD)

1. **Employment Status: Before COVID-19, were you…**

① Employed for wages

② Self-employed

③ Out of work and looking for work

④ Out of work but not currently looking for work

⑤ A homemaker

⑥ A student

⑦ Military

⑧ Retired

⑨ Unable to work

⑩ Other:_______________________________________________

1. **Employment Status: Are you currently…?**

① Employed for wages

② Self-employed

③ Out of work and looking for work

④ Out of work but not currently looking for work

⑤ A homemaker

⑥ A student

⑦ Military

⑧ Retired

⑨ Unable to work

⑩ Other:_______________________________________________

1. **Insurance status before COVID-19**

① Insurance through a current or former employer or union

② Insurance purchased directly from an insurance company

③ Medicare

④ Medicaid

⑤ TRICARE or other military health care

⑥ VA

⑦ Other: _______________________________________________

⑧ No health insurance

1. **Current Insurance**

① Insurance through a current or former employer or union

② Insurance purchased directly from an insurance company

③ Medicare

④ Medicaid

⑤ TRICARE or other military health care

⑥ VA

⑦ Other: _______________________________________________

⑧ No health insurance

1. **What is your race? (*select all that apply)***
   - White
   - Black or African-American
   - American Indian, Alaska Native, Native Hawaiian
   - Asian Indian
   - Asian:___________________________________
   - Pacific Islander: ___________________________
   - Other: ___________________________________
   - Prefer not to answer
2. **Are you of Hispanic, Latino, or Spanish origin?**
   - No, I am not of Hispanic, Latino, or Spanish origin
   - Yes, Mexican, Mexican American, Chicano
   - Yes, Puerto Rican
   - Yes, Cuban
   - Yes, another Hispanic, Latino or Spanish origin:_______

**Thank you for your time!**
